# Significance of Polarization Charges and Isomagnetic Surface in Magnetohydrodynamics

**DOI:** 10.1371/journal.pone.0136936

**Published:** 2015-08-31

**Authors:** Zhu-Xing Liang, Yi Liang

**Affiliations:** 18-4-102 Shuixiehuadu, Zhufengdajie, Shijiazhuang, Hebei, 050035, China; University of California San Diego, UNITED STATES

## Abstract

From the frozen-in field lines concept, a highly conducting fluid can move freely along, but not traverse to, magnetic field lines. We discuss this topic and find that in the study of the frozen-in field lines concept, the effects of inductive and capacitive reactance have been omitted. When admitted, the relationships among the motional electromotive field, the induced electric field, the eddy electric current, and the magnetic field becomes clearer. We emphasize the importance of isomagnetic surfaces and polarization charges, and show analytically that whether a conducting fluid can freely traverse magnetic field lines or not depends solely on the magnetic gradient along the path of the fluid. If a fluid does not change its density distribution and shape (can be regarded as a quasi-rigid body) and moves along isomagnetic surface, it can freely traverse magnetic field lines without any magnetic drag, no matter how strong the magnetic field is. Besides theoretical analysis, we also present experimental results to support our analysis. The main purpose of this work is to correct a fallacy among some astrophysicists.

## Introduction

The concept of frozen-in magnetic field originated from Alfvén [1] is often used in plasma physics and magnetohydrodynamics (MHD). This concept and the concept of conservation of magnetic flux are often referred to collectively as Alfvén’s Theorem. The concept of frozen-in field lines has been presented in various forms. A common interpretation is that a highly conducting fluid can move freely along, but cannot freely traverse, magnetic field lines. Because the frozen-in concept is of prime importance in MHD theory, it is widely mentioned in the literature [2, 3], and presented in many books of MHD and plasma physics [4–8]. Although the applicability of this concept has long been contested [3, 9], and Alfvén, the concept’s founder, also criticized its use and stated that it is often misleading [10, 11], it still plays an important role, specially in astrophysics. Here we present experimental and analytical evidence to support a conclusion that the concept of frozen-in field lines should not be used in MHD.

It has been established by Bellan that under certain conditions, a highly conducting fluid can freely cross magnetic field lines [12]. Here we develop and extend Bellan’s viewpoint.

## Analysis on the interaction between conducting fluid and magnetic field lines

For convenience, the term “fluid” used in the following means an ideal conducting fluid.

Although the concept of frozen-in field lines is often used by some authors, it currently lacks a specific definition. The following two definitions are familiar to many readers.

1. The magnetic flux through a fluid is conserved (we designate this definition “conservation theorem”).
2. Any motion of the fluid perpendicular to the field lines carries the field lines with the fluid (we designate this definition “frozen theorem”). This frozen theorem is often presented in another form: “a fluid cannot freely traverse magnetic field lines.”

Magnetic flux can be conserved in both scenarios:

1. All field lines within a fluid move along with the fluid.
2. The number of field lines entering a given fluid equals that leaving it at any instant.

Hence the two theorems are actually not equivalent. We accept the conservation theorem but question the frozen theorem.

There is a corollary to the frozen theorem: At low beta where the magnetic field is strong and produced by external coils, one pictures the fluid being pushed around by the magnetic field (so if the field is stationary, so is the fluid), while at high beta where the magnetic field is weak, one pictures the fluid pushing the magnetic field around. In either case, the magnetic field and the fluid move together. Note that this corollary and the frozen theorem include two notions:

1. Under the conditions that the external magnetic body and its magnetic field are stationary, if a fluid moves perpendicularly to the field lines, the field lines must bend and exert a resistive force on the fluid.
2. The magnitude of the resistive force exerted on the fluid depends on the magnetic field strength. The stronger the magnetic field, the greater the resistive force.

It is indisputable that in most cases when a fluid traverses a magnetic field, a resistive force will be exerted on the fluid. However, this does not mean that the traverse motion must result in the appearance of magnetic drag. In this paper, we seek those special conditions under which a fluid can freely traverse magnetic field without any magnetic drag, no matter how strong the magnetic field is.

### Condition for fluid’s freely traversing magnetic field lines

The frozen theorem follows from the evolution equation of the magnetic field (also referred to as frozen-in field equation)

$$\begin{array}{c} \frac{\partial B}{\partial t}=\nabla \times \left(u\times B\right), \end{array}$$ (1)

where **u** is the relative velocity of the fluid element moving through the magnetic field **B**, and **u** × **B** is the motional electromotive field. Eq (1) describes the evolution (namely turbulence) of the magnetic field with fluid motion traversing the magnetic field.

From Eq (1), we realize that

1. For any spatial position passed by a fluid, the magnetic evolution depends on the crossing motion of the fluid. Without crossing motion, there must be no magnetic evolution; and
2. Even a fluid crosses a magnetic field, namely **u** × **B** ≠ 0, as long as **∇** × (**u** × **B**) = 0, there must be no magnetic evolution.

This indicates that field-line crossing is a necessary, but not sufficient, condition for magnetic evolution. The magnetic evolution arising from the motion of the fluid always co-exists with magnetic drag and neither phenomenon can appear alone, because both arise from one and the same eddy current. Therefore, field-line crossing is also a necessary but not sufficient condition for magnetic drag. In other words, even if fluid crosses a magnetic field, i.e. **u** × **B** ≠ 0, the magnetic evolution and magnetic drag can be absent as long as the condition **∇** × (**u** × **B**) = 0 is satisfied.

It is known that the essential condition for a force acting between the fluid and the magnetic field is the presence of an eddy current within the fluid, whereas the essential condition for the presence of eddy current is the presence of an eddy motional electromotive field **u** × **B**. When both sides of Eq (1) are zero, the motional electromotive field within the fluid is irrotational. In this case, neither an eddy motional electromotive field nor an eddy current exists in the fluid. Consequently, no magnetic force is exerted on the fluid when it crosses a magnetic field with velocity **u**.

Now, the purpose to determine the condition under which a fluid can freely traverse magnetic field without magnetic drag turns into another one to determine the condition under which both sides of Eq (1) are equal to zero. To solve the latter problem, we expand Eq (1) as

$$\begin{array}{c} \frac{\partial B}{\partial t}=u\left(\nabla \cdot B\right)-B\left(\nabla \cdot u\right)-\left(u\cdot \nabla \right)B+\left(B\cdot \nabla \right)u. \end{array}$$ (2)

The first term on the right-hand side involves the divergence of the magnetic field **∇** ⋅ **B**, which, according to Maxwell’s equation, is always zero.

The factor **∇** ⋅ **u** in the second term is the divergence of the velocity field. If the density distribution of the fluid remains constant, this term also equals zero.

When each fluid element moves along an isomagnetic surface, the third term (**u** ⋅ **∇**)**B** vanishes.

If the fluid elements that synchronously cross the same magnetic field line have the same velocity (i.e., do not exhibit differential movement), the fourth term (**B** ⋅ **∇**)**u** vanishes too. In general, the differential movement is related to a change in shape of the fluid.

Eq (2) incorporates three essential factors that control the interaction between fluid and magnetic field:

1. The expansion or compression of the fluid, i.e., the change in density;
2. The differential movement of the fluid, i.e., the change in shape of the fluid; and
3. The change in magnetic field along the path of fluid’s movement.

These essential factors indicate that the so-called frozen-in phenomenon in reality relates to some changes. Without these changes, the frozen-in phenomenon cannot appear.

**Fig 1:**
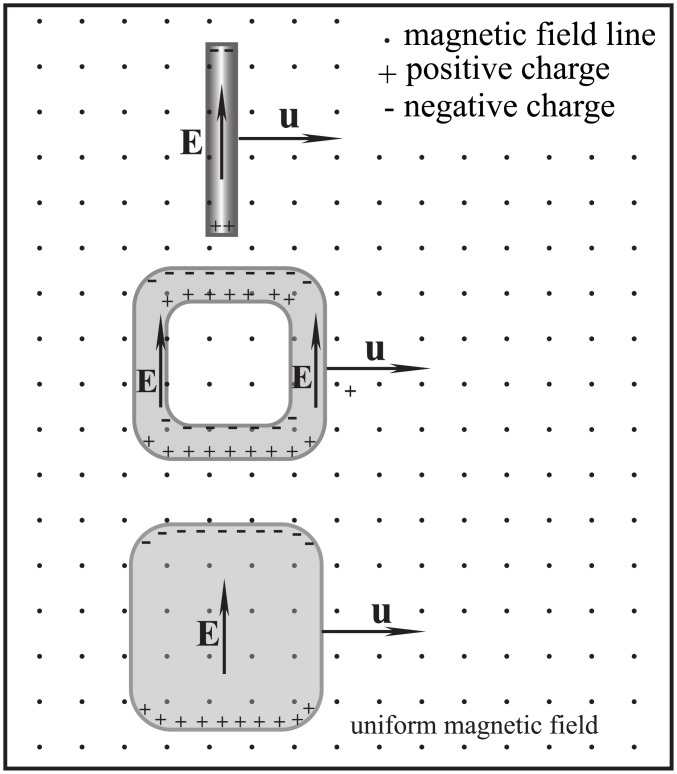
First non-frozen scenario. Moving across a uniform magnetic field, a fluid experiences no magnetic drag. Here, the distributions of the charges and electric fields have been simplified.

**Fig 2:**
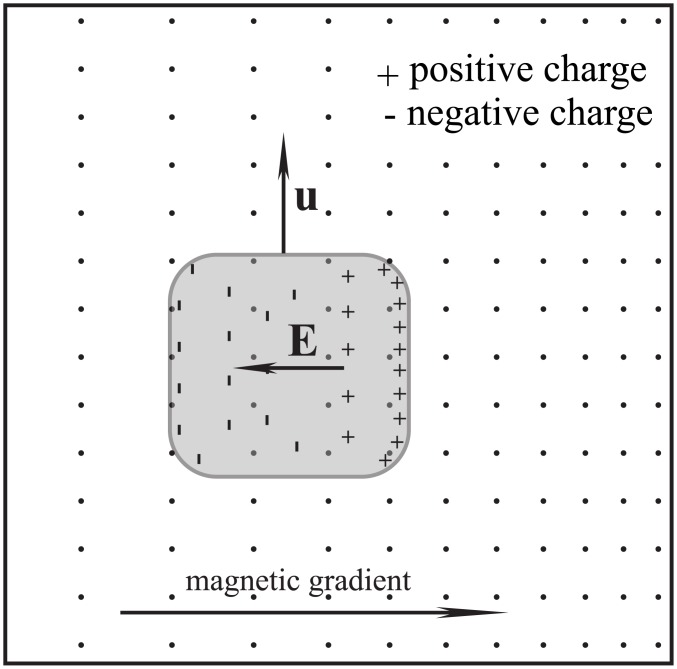
Second non-frozen scenario. When a fluid moves across non-uniform magnetic field, there is no frozen-in effect provided the fluid elements move along isomagnetic surfaces.

**Fig 3:**
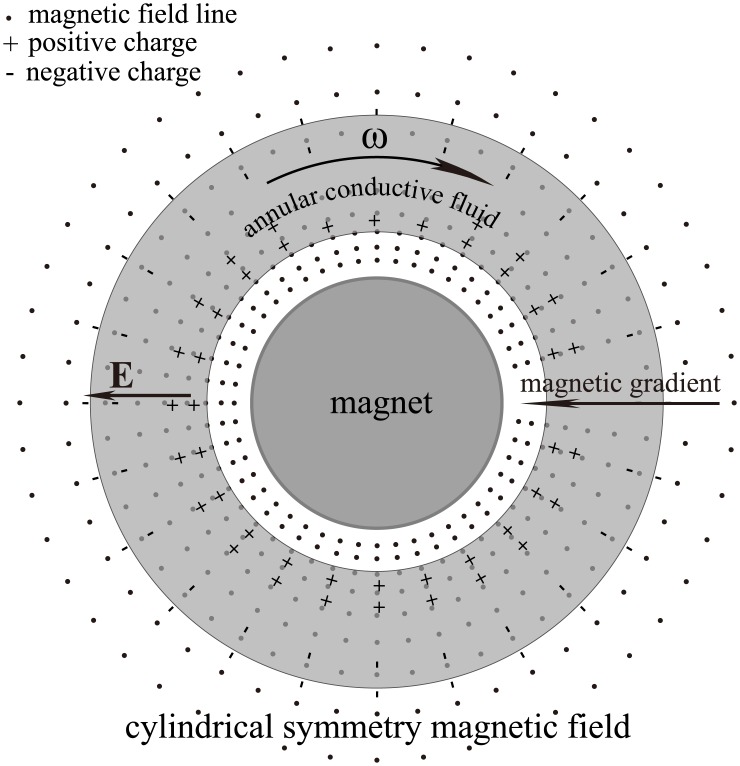
Third non-frozen scenario. In a cylindrically symmetric magnetic field, the fluid rotation around the magnetic axis would not result in frozen-in drag, even if the outer and inner fluids rotate at different angular speeds. This scenario mirrors the situation near the equatorial plane of aligned pulsar or the Sun.

If a fluid does not change its density distribution and shape, it can be regarded as a quasi-rigid body. The following simple law applies: *When crossing a magnetic field along isomagnetic surface, a rigid or quasi-rigid body will not be subject to any magnetic force, no matter how strong the magnetic field is and how fast the fluid moves.* This law fits the scenarios shown in Figs 1, 2 and 3. The three scenarios share two characteristics:

1. All of the fluid elements cross the magnetic field along isomagnetic surfaces.
2. The motional electromotive field **u** × **B** inside the fluid is everywhere nonzero but curl-free.

For the case shown in Fig 3, even if the outer and inner fluids rotate at different angular speeds, i.e. *ω* = *f*(*r*), no magnetic force acts on the fluid. However, if the differential rotation can be described as *ω* = *f*(*z*) (*z* is the axial coordinate), a magnetic force will act on the fluid. This distinction arises from the fourth term of the right-hand side of Eq (2). If *ω* = *f*(*r*), then (**B** ⋅ **∇**)**u** = 0 and no magnetic force appears; if *ω* = *f*(*z*), then (**B** ⋅ **∇**)**u** ≠ 0 and a magnetic drag will appear. This result is consistent with the law of iso-rotation (Ferraro’s theorem) which states that in the steady state, angular velocity is constant along magnetic lines [13]. If *ω* = *f*(*r*), the angular velocity varies only along the direction perpendicular to the field lines. In this situation, although the fluid is no longer quasi-rigid because of the appearance of differential rotation, it can still traverse magnetic field lines freely. This indicates that the constraint condition of “quasi-rigid” can be softened to a certain extent.

Alfvén & Fälthammar has described an example similar to that in Fig 1, for which the fluid can cross a uniform magnetic field under certain conditions [13]. Our work extends this idea by softening the constraint conditions for other cases of non-uniform magnetic field and/or differential rotation of the fluids.

Because the case shown in Fig 3 illustrates the situation in the vicinity of the equatorial plane of an aligned pulsar, we come to an important conclusion: the plasma near the equatorial plane of an aligned pulsar can cross the magnetic field and cannot be frozen with the magnetic field lines. In other words, the plasma can neither drive the magnetic field lines, nor be driven by the magnetic field lines. This conclusion is supported by the results of our magnetohydrodynamic experiments (see the section: Experiments on the isomagnetic surface).

Figs 1, 2 and 3 do not include all of the scenarios in which fluid can freely cross magnetic field lines. The conditions in the three scenarios merely ensure that all terms of the right-hand side of Eq (2) are zero. More generally, even if some terms are non-zero, so long as the terms sum to zero, the fluid can freely cross the magnetic field lines. For example, if a fluid element undergoes an apropos expansion rate, it can cross magnetic field lines against the direction of magnetic gradient.

**Fig 4:**
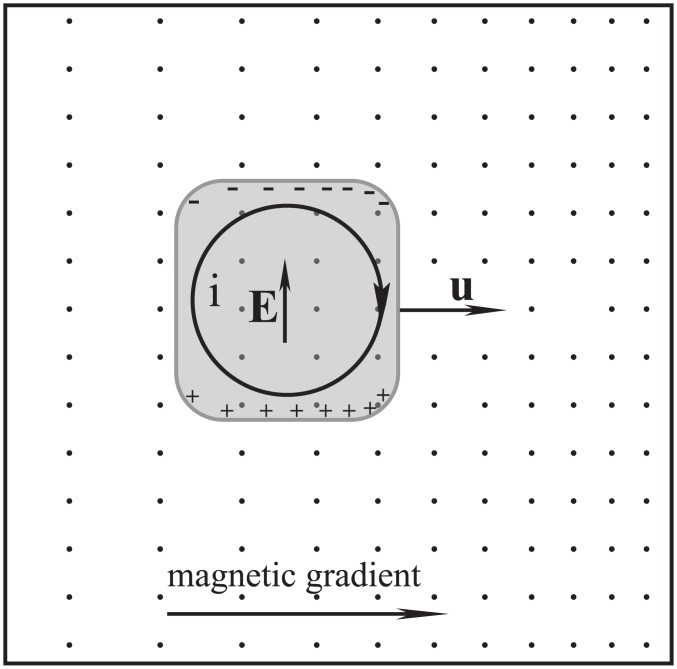
Magnetically frozen scenario. If the fluid velocity has a component in the direction of the magnetic field gradient, the fluid movement will result in a magnetic evolution and magnetic drag. The field lines caused by the eddy current were not illustrated.

Fig 4 shows a scenario in which the fluid experiences magnetic drag and results in magnetic evolution. When compare Fig 2 with Fig 4, the significance of isomagnetic surface can be seen clearly.

If a magnetic field changes in the direction of fluid movement, the fluid will generally experience drag. In particular, if the length of fluid along the direction of its motion is greater than the extent of the magnetic field, magnetic drag is inevitable at the magnetic boundary because the magnetic field changes sharply and the magnetic gradient is very high there.

Now, it has been clear that when a quasi-rigid fluid crosses a uniform magnetic field, the system is always force-free, no matter what velocity the fluid is moving with and how strong the magnetic field is. On the other hand, every steady magnetic field can be regarded as a superposition of uniform and non-uniform components and only the non-uniform component may, not must, result in magnetic drag. From this perspective, we can clearly understand the interaction between fluid and magnetic field and avoid to be misled by the frozen theorem.

### Effects of the polarization charges and polarization electrostatic field

The following equation describes the distribution of polarization charges inside the fluid:

$$\begin{array}{c} \frac{q}{\epsilon_{0}}=-\nabla \cdot \left(u\times B\right). \end{array}$$ (3)

In MHD literature, it seems that this equation is often belittled [14] even discarded from the MHD equations [15]. This might arise from the concept that conducting fluid is always electrically neutral anywhere inside a conducting fluid. In fact, the interaction between the fluid and the magnetic field can destroy the electrical neutrality and result in the appearance of surplus positive or negative charges inside the fluid. Especially, under the special conditions (such as Figs 1, 2 and 3), the fluid’s moving along the isomagnetic surface can result in neither magnetic disturbance nor magnetic force, but can result in electric field and surplus charges to appear in the systems. In these special cases, only the effects of the electric field and charges are noteworthy. Their importance can be shown by two examples. First, in the magnetosphere of pulsars, the density distribution of charges has been studied by Goldreich & Julian [16] and known by pulsar scientists as Goldreich-Julian density [17]. Second,the output voltage of magnetohydrodynamic generator is just caused by the induced electric field.

This demonstrates that in some cases, the electrical neutrality approximation must be abandoned and the effects of the electric field and charges must be considered.

According to conventional physics, a straight wire, closed wire or conducting plate (see Fig 1) moving perpendicularly in a uniform magnetic field can neither produce electric current nor experience drag from the magnetic field. Thus, the magnetic field lines were not considered to be frozen within the conductors. Because this phenomenon is independent of the type or conductivity of the materials, even if the objects shown in Fig 1 comprise fluid such as mercury or plasma, the frozen-in effect does not apply. However, in the MHD theory, the fluids in Fig 1 will be considered to be frozen in the magnetic field and cannot cross freely through the field lines. This presents an apparent contradiction between conventional physics and MHD theory.

In previous studies on the frozen-in effect, the effects of the polarization charges and electrostatic field **E** (all the electric field mentioned in this paper is the lab frame electric field) shown in Figs 1, 2 and 3 have been disregarded. In fact, it is the electric field **E** that produces a fluid drift and “thaws” the magnetic-frozen states. A simple calculation using the drift theory of charged particles [5] verifies that the drift velocity of both the ions and electrons produced by **E** and **B** is always equal to the macroscopic moving velocity of the fluid, i.e., **v**_drift_ = (**E** × **B**)/*B* ^2^ ≡ **u** [18, 19].

Bellan [12] has demonstrated that a plasma immersed in a static magnetic field **B** and a static electric field **E**, with **E** perpendicular to **B**, will freely drift across the magnetic field lines. Based on Bellan’s viewpoint, we claim that the static electric field **E**, which is established by polarization charges, can naturally appear under certain conditions. The process by which this can occur is summarized below:

- A fluid moving across the magnetic field lines induces an irrotational motional electromotive field **u** × **B** inside the fluid;
- As a result of this irrotational motional electromotive field, polarization charges appear inside the fluid or its boundaries;
- The polarization charges result in a static polarization electric field, and the motional electromotive field is balanced by the polarization electric field, **E** = −**u** × **B**;
- **E** combines with **B** to “force” all the particles (ions and electrons) to drift with velocity **v**_drift_ equal to **u**. Namely, the velocity of the guiding-center drift equal to the motion velocity of the fluid.

Finally, the system is force-free and the fluid can move freely as the magnetic field is absent.

When an individual charged particle travels through a magnetic field, it is subject to Lorentz forces which compel it to gyrate around the magnetic field lines. However, within a fluid, the presence of both polarization charges and electric field create a very different situation. Under some special conditions, such as those illustrated in Figs 1, 2 and 3, the static electric field produced by the polarization charges can counteract the Lorentz force and the fluid elements will drift across the magnetic field lines without any drag.

In the situations shown in Figs 1, 2 and 3, the relative velocity between the fluids and the field lines cannot be determined by magnetism method, because the magnetic fields have no evolution. However, it is incorrect to think the relative velocity is arbitrary, even zero (frozen-in), because the relative velocity can be determined by electrical method. For example, by measuring the output voltage of a magnetohydrodynamic generator, the relative velocity can be determined.

### Infinite conductivity vs finite current

There is an opinion in some books [5–7] whose typical description is following [8]:

Because the conductivity of ideal conducting fluid is infinite, if the induced electric field appears inside the fluid, the induced current must be infinite. Hence the induced electric field cannot appear inside the ideal conducting fluid.

Their opinion is incorrect and the root of this mistake is that the Ohm’s law (*I* = *V*/*R* or **J** = *σ* **E**) was incorrectly used to the non-steady or open circuit system.

From electrical engineering we know that in a non-steady circuit system, beside resistance, inductive and capacitive reactance can also restrain the current growth. In other words, it is the complex impedance (not only the resistance) that controls the current growth. This law is also valid in MHD field because the electric field, current, and magnetic field are generally time-variant. It is similar that in the DC circuit, only resistance is considered, but for the AC circuit, the reactance must be considered. Below, we show how the inductive reactance and capacitive reactance restrain the current growth and why the fluid can traverse magnetic field lines.

When a fluid traverses magnetic field lines, a motional electromotive field **u** × **B** must appear to drive a current to grow. At the same time, an induced electric field **E** immediately appears to restrain the current growth and makes the current always finite. The relation between them is **E** = −**u** × **B**. Since the motional electromotive field **u** × **B** has two components, rotational and irrotational components, the induced electric field **E** has two components too.

The rotational component of the motional electromotive field is **∇** × (**u** × **B**). The opposite component of the induced electric field is **∇** × **E** = −∂**B**/∂*t*. This induced electric field component is caused by the time-variant magnetic field. The time-variant magnetic field is caused by the time-variant eddy current and this eddy current is caused by the rotational motional electromotive field. As long as the direction of **∇** × (**u** × **B**) does not reverse, the eddy current will grow continually. But the rate of growth depends on the inductive reactance. In a real system, the magnetic field caused by the eddy current only offsets the change of the external magnetic field along the motion path and conserves the magnetic flux; so the eddy current must be finite unless the external magnetic field can become infinitely strong. From that, it is the inductive reactance that restrain the eddy current to be always finite inside the ideal conducting fluid.

When a rotational electric field appears inside an ideal conducting fluid, the system must be evolutive. The Ohm’s law cannot be used in such non-steady system.

The irrotational component of the motional electromotive field is **∇** ⋅ (**u** × **B**) and the opposite component of induced electric field is **∇** ⋅ **E** = *q*/*ϵ*_0_. This induced electric field component is caused by the polarization charges and the polarization is driven by the irrotational motional electromotive field. As long as a fluid traverses a magnetic field, this irrotational component of the induced electric field is always non-zero as shown in Figs 1–4. Because relating usually to slow variation the capacitive reactance is very high, so that the capacitive current can usually be ignored. Namely, it is the capacitive reactance that restrain the irrotational current. For the isolated fluids as shown in Figs 1, 2 and 3, we can regard them as batteries (but the chemical electromotive force is replaced by the motional electromotive force) without external circuit. The Ohm’s law cannot be used in such open circuits.

However, the boundary conditions can affect the irrotational current. In the cases shown in Figs 1, 2 and 3, there are irrotational electric fields but no current. Now we assume to add an external wire connected to the two boundaries where the electrons accumulated. Will an electric current appear? There are two cases: First, if the external wire moves or rotates with the fluid and cuts the field lines, the induced electric field inside the fluid will counteract the one inside the wire and no current will appear; Second, if the external wire is stationary, induced current and magnetic drag will appear—this is just the principle of magnetohydrodynamic generator and homopolar generator. Significantly, even if in the second case, the induced electric field **E** and the motional electromotive field (**u** × **B**) are still nonzero.

Inside the conducting fluid (shaded area in Figs 1–4), it is correct to think always **E**+(**u** × **B**) = 0; but it is wrong to think always **E** = 0 and **u** × **B** = 0. When taking the inductive and capacitive reactances into account, we can understand why **E** ≠ 0 and **u** × **B** ≠ 0 would not result in an infinite current inside ideal conducting fluid.

From the above analyses, we can get following logical relation: If a fluid cannot traverse magnetic field lines, the motional electromotive field **u** × **B** must be equal to zero. If **u** × **B** ≡ 0, the induced electric field **E** must be equal to zero. If **E** ≡ 0, then ∂**B**/∂*t* ≡ 0 and *q* ≡ 0. In this manner, the evolution equations ∂**B**/∂*t* = −**∇** × (**u** × **B**) and *q* = *ϵ*_0_ **∇** ⋅ (**u** × **B**) will be meaningless.

All in all, the non-zero motional electromotive field caused by the fluid’s traversing motion is the root of the system evolution and would not result in an infinite current. Supposing that the fluid freezes with the field lines and deny the field-line crossing, we will actually deny the system evolution. Therefore we have to accept the concept that a conducting fluid can traverse magnetic field lines. The field-line crossing is not only acceptable but also indispensable.

### Problems arising from the frozen theorem

We have discussed that the fluid elements can freely cross magnetic field lines in the cases of the uniform magnetic field and quasi-rigid fluids. Other researchers [20], however, demonstrated that if the fluid elements are fixed on the magnetic field lines and move always together, Eq (1) is equally satisfied. Some researchers thus advocated that both choices are valid [21]: 1) the fluid moves across magnetic field lines; and 2) the fluid and the magnetic field lines move together (i.e. frozen theorem). However, following careful analysis, some problems will arise from the frozen theorem.

**Fig 5:**
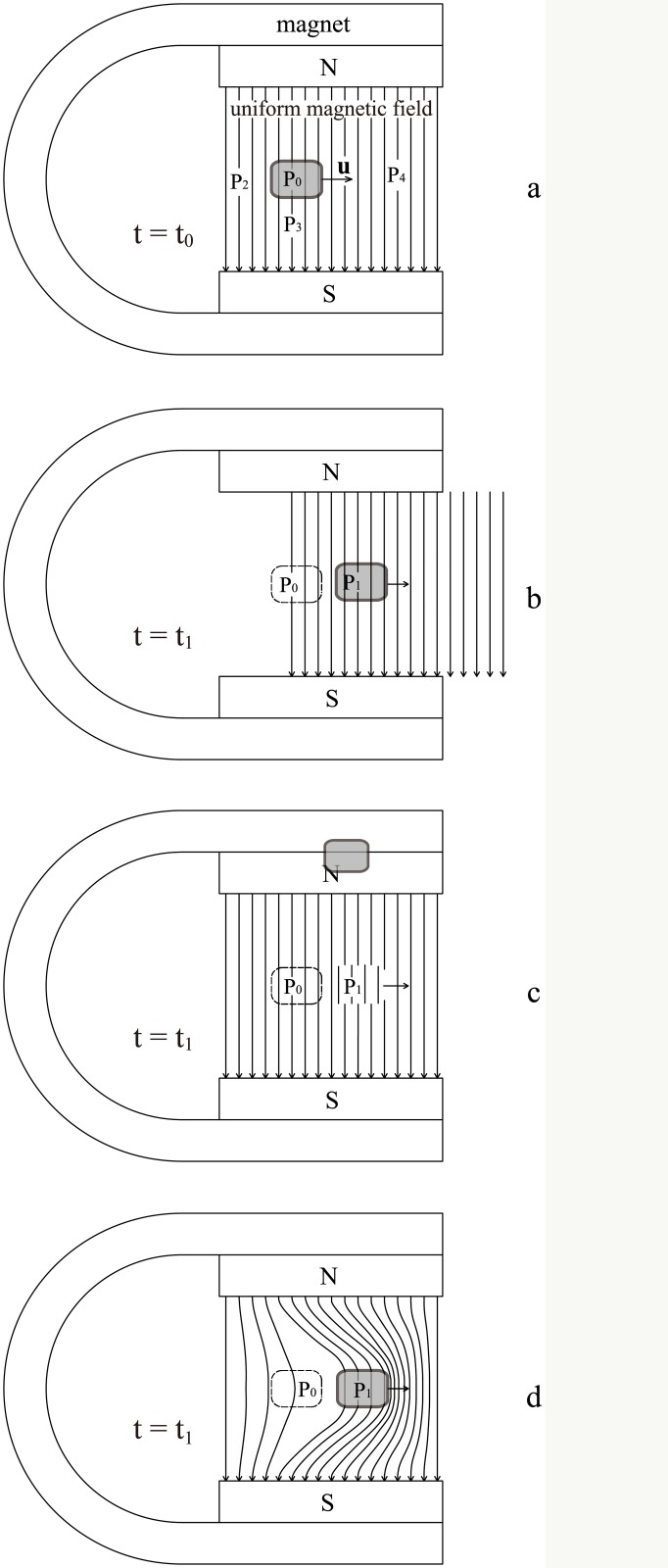
Different scenarios for the magnetic evolution outside a fluid. (a) The fluid will move across the uniform magnetic field. (b) If the fluid can carry the field lines inside and outside the fluid with the same velocity, the field lines will translate and retain their shape. (c) If the fluid can carry only the field lines inside the fluid, the field lines will be repeatedly cut off and reconnected at the fluid boundary. (d) If the fluid can carry the field lines inside the fluid and all field lines remain continuous, the field will become non-uniform.

In previous discussions on the frozen theorem, attention has usually been devoted to only the fluid and the magnetic field lines inside the fluid. However, as long as the system studied is extended to also include the external magnet and the magnetic field lines outside the fluid (see Fig 5), we will see that contradictions are unavoidable.

First, we assume that the magnetic field is uniform, the magnet is immobile and the quasi-rigid fluid moves with velocity **u** relative to the magnet, as shown in Fig 5a. Then we consider the changes of the magnetic field lines outside the fluid.

If the field lines outside the fluid respond to the fluid motion, only three possible solutions can be considered, as illustrated in Fig 5b, 5c and 5d.

1. In Fig 5b, all magnetic field lines outside the fluid translate with the fluid. As the velocities are identical everywhere, the configuration of the field lines cannot change and the field lines must be cut off at the boundary of the magnet. Apparently, this solution is unacceptable.
2. In Fig 5c, all the magnetic field lines outside the fluid are stationary relative to the magnet. In this scenario, the field lines must be cut off by the fluid boundary and must repeatedly reconnect. However, this result contradicts the alternative representation of the frozen theorem: “Any fluid element that is at one instant on a magnetic field line will be on the original magnetic field line at any other instant”. Because a reconnected magnetic field line is not the original one, then the fluid cannot remain attached to the original magnetic field lines. If we accept that the magnetic field lines can be cut off, we have actually changed the frozen theorem to that *any field-lines passing through fluid will be cut off by fluid’s crossing motion.* Apparently, this solution is also unacceptable.
3. In Fig 5d, the magnetic field lines outside the fluid are dragged by the fluid and change the magnetic field configuration. However, this configuration induces a magnetic evolution and magnetic drag, and is inconsistent with the analysis above. Therefore, it should be abandoned, too.

Some problems arisen from the misuse of the concept of moving field lines have been discussed by other researchers [18, 22]. With the help of our new visual angle and the analyses above, we conclude that, as long as the system studied is extended and the magnet and its field lines outside the fluid are also included, the frozen theorem always leads to contradictions.

### Better interpretation than the frozen theorem

**Fig 6:**
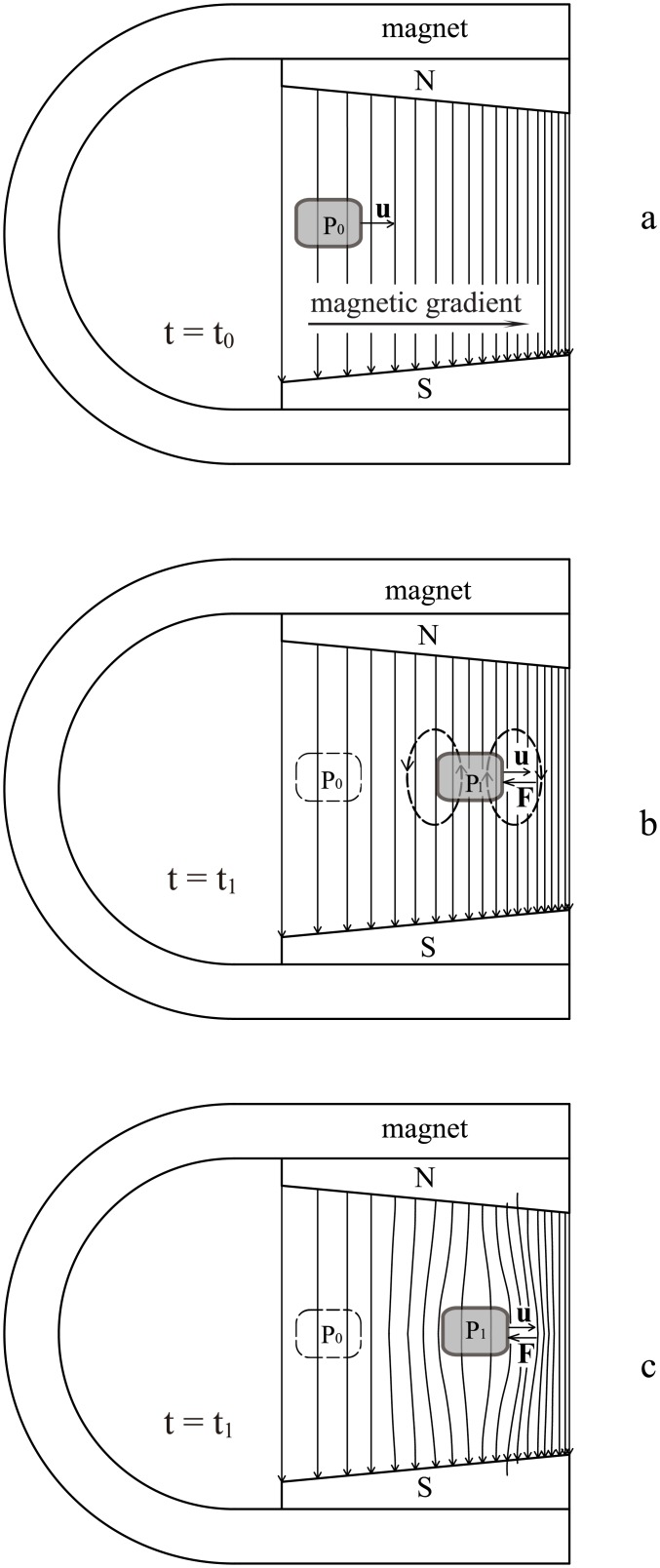
Two-magnet interpretation for the interaction between fluid and magnetic field lines. When moving from P_0_ to P_1_, the fluid becomes a magnet, as shown in panel b. When the two magnetic fields in panel b are combined, the magnetic configuration can be illustrated as panel c.

We suggest using a better method to describe the relationship between moving fluid and magnetic field lines. When a fluid crosses a magnetic field along the isomagnetic surface, we can consider that the field lines are stationary with the magnet and the fluid crosses them freely. If a fluid crosses a non-uniform magnetic field along the direction of magnetic gradient, as shown in Fig 6a, we can still consider that the field lines caused by the external magnet are stationary, but the magnetic gradient creates an eddy current inside the fluid and the fluid has become a fluidic magnet. The field lines associated with this fluidic magnet are shown with dashed lines in Fig 6b.

The magnetic drag is, in fact, the force acting between the external magnet and the fluidic magnet. Although the forces acting on the front and rear sides of the fluid are always opposite, the magnetic gradient makes their magnitudes different. No matter what direction the fluid is moving, parallel or opposite to the magnetic gradient, the result of the forces is always opposite to the motion direction.

When the two magnetic fields are combined to form one field, Fig 6b becomes Fig 6c. The magnetic flux in the fluid remains constant but the magnetic configuration outside the fluid has changed compared with Fig 6a.

A notable point is that the dashed field lines at P_1_ in Fig 6b are produced in P_1_ instead of pulled from P_0_, because these dashed magnetic field lines had not appeared when the fluid was at P_0_. Therefore, this two-magnet interpretation needs neither frozen-in field lines nor moving field lines.

Under the condition that the fluid is a quasi-rigid body, this two-magnet interpretation infers the following:

1. When a fluid crosses magnetic field lines, eddy current can appear inside the fluid.
2. The eddy current turns the fluid into a fluidic magnet and causes an additional magnetic field to appear.
3. Because the two-magnets interact with each other, the magnetic drag appears.
4. The additional magnetic field always counteracts changes in magnetic flux caused by the external magnet and conserves the total flux inside the fluid.
5. The additional magnetic field will change the magnetic configuration outside the fluid.
6. Because the eddy current depends on the magnetic gradient, the effects above disappear outright if the fluid moves along the isomagnetic surface.

Compared with the frozen theorem, this two-magnet interpretation has the following strengths:

1. Using the two-magnet interpretation, we can clearly understand not only the magnetic conservation inside the fluid but also the magnetic variation outside the fluid.
2. The concept of magnetic reconnection has no longer been needed, even on the fluid boundary.
3. The problems shown in Fig 5b–5d can be avoided altogether.
4. Since this two-magnet interpretation emphasizes that the eddy current depends on the magnetic gradient, the importance of the isomagnetic surface become easier to understand.
5. Since this two-magnet interpretation emphasizes the field-lines crossing, it comes clear that the fluid motion causes the volume density distribution of the polarization charges.

Note that this interpretation is very different with the frozen theorem. As shown in Fig 6c, when the fluid moves from P_0_ to P_1_, the field lines to the left of P_1_ grow denser instead of sparser. If the frozen theorem holds and field lines have been carried by the fluid from P_0_ to P_1_, the field lines there must become sparser and are similar to Fig 5d. Similarly, when the fluid moves left, ahead of the fluid, the field lines do not become denser; quite the reverse, they become sparser. This indicates that this two-magnet interpretation is not compatible with the frozen theorem. In other words, from the two concepts, we cannot derive identical evolution results for the magnetic field lines outside the fluid. Therefore, it is impossible maintain the two concepts concurrently and one must be abandoned.

### Summary contrasts in tables

In Table 1, we contrast two cases and clarify the relationship between the electromagnetic phenomena and Eq (1).

**Table 1:** Contrast between two cases of Eq (1).

	case A	case B
both sides of Eq (1)	= 0	≠ 0
property of magnetic field	steady magnetic field	time-varying magnetic field
eddy current in fluid	absence	presence
magnetic drag	absence	presence
illustration	Figs 1, 2 and 3	Fig 4

For clarity, the analysis results of Eqs (1) and (3) are summarized in Table 2. Previous studies have mainly focused on those properties listed in column A and have overlooked all of the aspects listed in column B; that is, the frozen-in effect has been emphasized whereas the drift effect has been largely ignored.

**Table 2:** Summary of the analysis results of Eqs (1) and (3).

	magnetic freezing (column A)	magnetic “thaw”(column B)
correlative equations	∂**B**/∂*t* = **∇** × (**u** × **B**)	*q*/*ϵ*_0_ = −**∇** ⋅ (**u** × **B**)
motional electromotive field parameter	curl	divergence
induction phenomena	inductive eddy current	polarization charges and electric field
correlative magnetic field parameter	magnetic field gradient	magnetic field strength
direction of fluid movement	parallel to magnetic gradient	perpendicular to magnetic gradient
effect of magnetic force	resisting the traversing motion	inducing field-line traversing
illustration	Fig 4	Figs 1, 2 and 3

The frozen theorem was expressed in several forms, which are summarized in Table 3. All of the expressions listed in the right-hand column are derived from the frozen theorem, and hence are incorrect or incomplete.

**Table 3:** Summary of the expressions of the frozen theorem.

correct expressions	incorrect or incomplete expressions
The magnetic flux in any fluid element is conserved.	Any motion of fluid, perpendicular to the field lines, carries the field lines with the fluid.
Any two elements of the same fluid that are at one instant on a common magnetic field line will be on a common magnetic field line at any other instant [14, 23].	Any fluid element that is at one instant on a magnetic field line will be on the original magnetic field line at any other instant.
Quasi-rigid fluid can freely traverse magnetic field lines only along the isomagnetic surface.	A fluid cannot freely traverse magnetic field lines.

## Experiments on the isomagnetic surface

**Fig 7:**
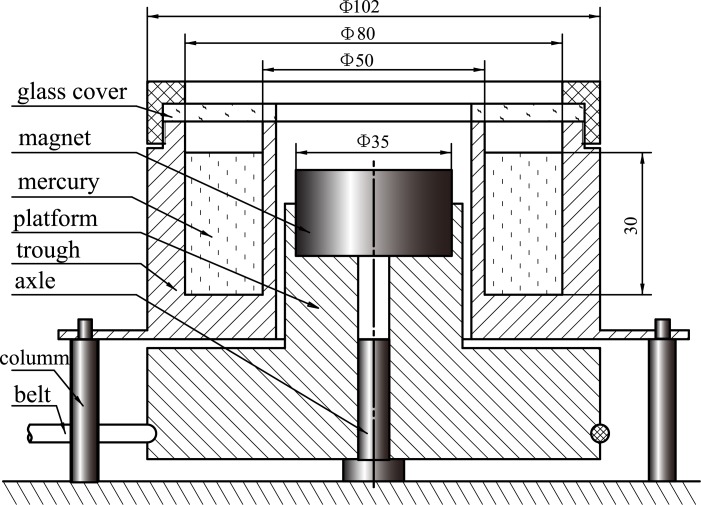
Schematic figure of drive experimental device. The unit of length is millimeter. The belt drives the rotation of the platform and magnet; the other components are stationary.

**Fig 8:**
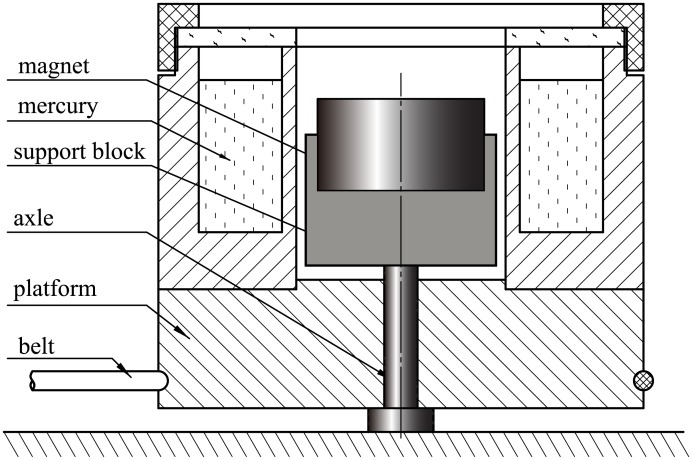
Schematic figure of braking experimental device. The axis, support block, and the magnet are stationary. The belt drives the rotation of other parts.

To verify the special significance of isomagnetic surface, we conducted a series of magnetohydrodynamic experiments. The configuration of the device is the same as that of Fig 3. For simplicity and clarity, the experiments were recorded and the videos can be viewed on YouTube or Tudou (for Chinese). Figs 7 and 8 depict the device configurations and the sizes of the main components. Apart from the magnet, all components are made of non-magnetic material (plastic or copper). The mercury was placed in a cylindrical trough surrounding a rotating platform. At the magnet surface, the magnetic field intensity is about 0.6 T. The rotational rates are shown in the relevant videos.

The experiments were divided into two groups. The first group comprised three pairs of experiments to determine the conditions under which the magnet can drive rotation of the mercury. In each experiment the basis of comparison is an *aligned magnetic rotator*, a cylindrically symmetric magnet whose magnetic axis aligns with its rotation axis. The three experiments are as follows:

1. *Drive experiment A* [24]. Revealed the contrast between the aligned rotator and a rotator with magnetic axis orthogonal to the rotation axis. This contrast test indicates that the transverse magnetic field can drive the mercury to rotate, but the aligned magnetic field cannot do that.
2. *Drive experiment B* [25]. Revealed the contrast between the aligned rotator and a magnet with magnetic axis parallel to, but displaced from the rotation axis. This contrast test indicates that the eccentricity magnetic field can drive the mercury, but the coaxial magnetic field cannot do that.
3. *Drive experiment C* [26]. Revealed the contrast between the aligned rotator and a magnet that is not cylindrically symmetric. This contrast test indicates that the cylindrically asymmetric magnetic field can drive the mercury, but the cylindrically symmetric magnetic field cannot do that.

The first group of experiments demonstrated that an aligned magnetic rotator cannot drive the mercury to rotate. However, this result has not proved the absence of the magnetic frozen effect in such cases, since this result can be attributed to another cause: the magnetic field lines don’t co-rotate with the aligned magnetic rotator. When a magnet rotates around its own magnetic axis, whether the magnetic field corotates with the magnet has been a controversial issue. This issue is also named as Faraday paradox. Although most of astrophysicists think the magnetic field corotates with the magnet, we take the opposite point of view because it is accepted by the most of general physical scientists and supported by more experiments for Faraday paradox.

Fälthammar once pointed that field lines are not tangible objects that move in the normal sense. One cannot distinguish the axisymmetric field of a cylindrical magnet rotating about its axis from the field of a non-rotating magnet and so one cannot assign a rotation to the magnetic field lines [18]. Backus also pointed that no effect appears outside an aligned magnetic rotator [27]. Hence, the result cannot prove the absence of the magnetic frozen effect in the experiments above.

The value of these experiments is to answer the question: Can an aligned pulsar drive its plasma and achieve the co-rotation state? The answer is NO.

In the second group of experiments, the conditions under which rotating mercury can be slowed by a stationary magnet were determined. The mercury trough (rather than the central magnet) was spun until the mercury revolution reached a steady state. In each experiment the basis of comparison was an *aligned magnet*, a cylindrically symmetric magnet whose magnetic axis matched that of the mercury rotation axis. In this case, the mercury eventually revolved at the same rate as the trough. For the three comparison cases, braking forces reduced the rotation of the mercury. We used the same magnets in every pair of braking experiments, designated A, B and C and described below.

1. *Braking experiment A* [28]. Revealed the contrast between the aligned magnet and the orthogonal magnet. This contrast test indicates that the orthogonal magnetic field can slow down the mercury, but the aligned magnetic field cannot do that.
2. *Braking experiment B* [29]. Revealed the contrast between the aligned magnet and the off-axis magnet. This contrast test indicates that the eccentricity magnetic field can slow down the mercury, but the aligned magnetic field cannot do that.
3. *Braking experiment C* [30]. Revealed the contrast between the aligned magnet and the cylindrically asymmetric magnet. This contrast test indicates that the cylindrically asymmetric magnetic field can slow down the mercury, but the cylindrically symmetric magnetic field cannot do that.

The braking experiments demonstrated that even if the magnetic field is non-uniform, fluid can experience no frozen-in effect, as long as all of the fluid elements move along isomagnetic surface. Thereby, the analyses for Fig 3 have been corroborated by our experimental results.

These experimental results have verified the special significance of isomagnetic surface: Along isomagnetic surface, a magnetic field can neither drive nor brake conducting fluid.

## Discussion

Our ideas are embodied in the following quote from Feynman et al. [31]: “It makes no sense to say something like: When I move a magnet, it takes its field with it, so the lines of **B** are also moved. There is no way to make sense, in general, out of the idea of ‘the speed of a moving field line.’”

In fact, a magnetic field essentially cannot be ascribed a movement. Time-varying magnetic fields exist, whereas moving ones do not. While it can be intuitive and useful to regard magnetic field lines as moving, this approach is often unsafe as discussed by Fälthammar & Mozer [18].

In conventional physics, certain electromagnetic problems have been correctly solved by assuming moving field lines. But in those situations, the magnetic poles always move with the field lines. By contrast, typical discourses on the frozen theorem did not clarify whether the magnetic source body is in motion or at rest. If the external magnet is at rest (generally the default condition), the assumption that the field lines are movable always makes the physics more complex and even leads to wrong results. Herein lies the root of the problem caused by the frozen theorem.

For some astrophysical objects, the magnetic field may be very strong, but the magnetic gradient in the direction of the plasma movement is usually very small, especially for the cases similar to that shown in Fig 3. Consequently, the effect of magnetic drag should be much weaker than that expected and the plasma can in fact freely cross the magnetic field lines. In other words, in some astrophysical objects, the magnetic frozen-in effect can be ignored provided that the magnetic gradient along the path of plasma movement can be ignored.

Magnetic confinement technology has been widely applied in nuclear fusion engineering. In those devices, plasma is restrained in the magnetic beam and cannot move laterally. One interpretation for this phenomenon is that the very strong magnetic field restrains the plasma. Actually, it is the transverse magnetic gradient that does the restraining, especially near the boundary surface of the magnetic beam. Any uniform magnetic field, regardless of strength, cannot restrain a conductive substance. But some people think that at low beta where the magnetic field is strong, the fluid will be pushed around by the magnetic field lines and if the field is stationary, so is the fluid. The reason why some people think so may be that they were misled by the frozen theorem.

When the magnetic evolution and magnetic drag are the study objects, the electrical neutrality is indeed an excellent approximation. However, when the drift of fluid particles and fluid’s crossing motion are concerned, we must pay more attention to the electric field and the charges distribution. In the latter case the electrical neutrality approximation is unacceptable.

Whether a quasi-rigid conducting fluid can freely traverse magnetic field lines or not depends solely on the magnetic gradient along the fluid path. No matter how strong the magnetic field is or how fast the fluid speed is, a fluid can freely traverse the magnetic field lines as long as it moves along the isomagnetic surface. Especially, no matter how strong the magnetic field is, a uniform magnetic field cannot push around conducting fluid.

The literal meaning of “frozen-in” is liable to promote an erroneous association between fluid traversing motion and magnetic drag. Therefore, we suggest that the use of this term should be avoided whenever possible. In particular, the frozen theorem should not be used to study MHD problems.
